# Short- and long-term follow-up of patients with non-neoplastic esophageal perforation

**DOI:** 10.1007/s00423-021-02327-1

**Published:** 2021-09-25

**Authors:** Sebastian Brinkmann, Laura Knepper, Hans Fuchs, Arnulf Hoelscher, Kathrin Kuhr, Daniel Pinto dos Santos, Patrick Plum, Seung-Hun Chon, Christiane Bruns, Wolfgang Schroeder, Jessica Leers

**Affiliations:** 1 Department of General, Visceral, Cancer and Transplantation Surgery, University of Cologne, Kerpener Str. 62, 50937 Cologne, Germany; 2 Department of General, Visceral and Vascular Surgery, Marien Hospital Herne, Ruhr University Bochum, Hölkeskampring 40, 44625 Herne, Germany; 3 Department of General, Visceral and Trauma Surgery, Elisabeth-Krankenhaus Essen, Essen, Germany; 4 Institute of Medical Statistics, Informatics and Epidemiology, University of Cologne, Cologne, Germany; 5 Department of Radiology, University of Cologne, Cologne, Germany

**Keywords:** Non-neoplastic esophageal perforation, Health-related quality of life, Long-term follow-up, Endoscopic endoluminal stent placement, Endoscopic vacuum therapy, Transthoracic esophagectomy

## Abstract

**Purpose:**

Esophageal perforation is associated with high morbidity and mortality. In addition to surgical treatment, endoscopic endoluminal stent placement and endoscopic vacuum therapy (EVT) are established methods in the management of this emergency condition. Although health-related quality of life (HRQoL) is becoming a major issue in the evaluation of any therapeutic intervention, not much is known about HRQoL, particularly in the long-term follow-up of patients treated for non-neoplastic esophageal perforation with different treatment strategies. The aim of this study was to evaluate patients’ outcome after non-neoplastic esophageal perforation with focus on HRQoL in the long-term follow-up.

**Methods:**

Patients treated for non-neoplastic esophageal perforation at the University Hospital Cologne from January 2003 to December 2014 were included. Primary outcome and management of esophageal perforation were documented. Long-term quality of life was assessed using the Gastrointestinal Quality of Life Index (GIQLI), the Health-Related Quality of Life Index (HRQL) for patients with gastroesophageal reflux disease (GERD), and the European Organization for Research and Treatment of Cancer (EORTC) questionnaires for general and esophageal specific QoL (QLQ-C30 and QLQ-OES18).

**Results:**

Fifty-eight patients were included in the study. Based on primary treatment, patients were divided into an endoscopic (*n* = 27; 46.6%), surgical (*n* = 20; 34.5%), and a conservative group (*n* = 11; 19%). Short- and long-term outcome and quality of life were compared. HRQoL was measured after a median follow-up of 49 months. HRQoL was generally reduced in patients with non-neoplastic esophageal perforation. Endoscopically treated patients showed the highest GIQLI overall score and highest EORTC general health status, followed by the conservative and the surgical group.

**Conclusion:**

HRQoL in patients with non-neoplastic esophageal perforation is reduced even in the long-term follow-up. Temporary stent or EVT is effective and provides a good alternative to surgery, not only in the short-term but also in the long-term follow-up.

## Background

Despite recent progress in surgical and endoscopic therapy, non-neoplastic esophageal perforation is still associated with high morbidity and mortality [1]. Various surgical, endoscopic, and conservative treatment strategies are available [2, 3]. Important for the outcome and for treatment choice is the extent of wound cavity, time of diagnosis, clinical inflammatory response, localization, and cause of perforation. There is a high variety of causes of esophageal perforation including spontaneous perforation, namely Boerhaave syndrome, iatrogenic perforation due to routine diagnostic endoscopy or transesophageal ultrasound, and rare traumatic causes. This variety leads to wide heterogeneity of treatment modalities resulting in an individualized treatment strategy for each patient based on cause of perforation. Over the last decades, treatment of esophageal perforation has changed from surgical treatment with diversion to more interventional treatment with stenting or endoscopic vacuum treatment.

Several studies have reported promising results in the short-term follow-up after effective treatment of this devastating complication [4, 5]. However, data on long-term follow-up are rare and especially HRQoL remains unclear. This study reports early outcome and efficacy of current treatment modalities and investigates patients’ outcome after non-neoplastic esophageal perforation with focus on HRQoL in the long-term follow-up. Furthermore, the study aims to analyze the association between long-term outcome and different treatment modalities.

## Methods

### Patients and data collection

Patients who were treated for non-neoplastic esophageal perforation at the University Hospital Cologne between January 2003 and December 2014 were included in this study. They were identified from a prospectively maintained database, and outcome data were recorded in a phone- and/or mail-based interview using the Health-Related Quality of Life (HRQoL) questionnaires. At the onset of the study, 25 of 58 (43.1%) patients were already deceased. Of the remaining 33 patients, a total of 20 were available for the survey.

Diagnosis was established mainly by CT imaging and endoscopy. Initial diagnostic procedure was predominantly based on etiology and site of esophageal perforation. Primarily established therapeutic management was based on the cause, site and size of perforation, and on overall health status of the patient and signs of sepsis. In sum, diagnostic and therapeutic management did not follow a standardized protocol and were individualized for each patient. Demographic and clinical data of patients were collected, including patient characteristics; time and date of diagnosis; cause, site, and length of esophageal perforation; and treatment specifics. Peri- and postoperative complications, length of ICU and hospital stay, and mortality were analyzed. The protocol of this study was approved by the Ethics Committee of the University Hospital of Cologne (reference number 16–268).

### Health-related quality of life assessment

HRQoL was assessed using four questionnaires: the Health-Related Quality of Life Index for GERD, the Gastrointestinal Quality of Life Index (GIQLI), and the EORTC QLQ-C30 and QLQ-OES18 [6–8].

HRQoL for GERD contains 11 items. Ten of the items ask for symptoms and are scored from 0 to 5, with a higher score indicating more severe symptoms. One item asks for satisfaction with the patients’ present condition which can be stated as satisfied, neutral, or unsatisfied.

The GIQLI measures quality of life in patients with gastrointestinal diseases. It includes 36 items with scores ranging from 0 to 4, resulting in an overall score between 0 and 144. Scores can also be divided into five domains: symptoms, emotional function, physical function, social function, and effect of medical treatment. Higher scoring represents a better HRQoL.

Additional HRQoL data were generated with the EORTC QLQ-C30 (version 3.0) questionnaire and the esophageal cancer specific module QLQ-OES18. The QLQ-C30 was developed to measure HRQoL in cancer patients and consists of 30 items. The items can be combined to assess five functional scales (physical, role, cognitive, emotional, social), nine symptom scales (fatigue, nausea/vomiting, pain, dyspnea, insomnia, appetite loss, constipation, diarrhea, financial difficulties), and one global health status scale. QLQ-OES18 includes nine symptom scales (eating, reflux, pain, trouble swallowing saliva, choked when swallowing, dry mouth, trouble with taste, trouble with coughing, trouble talking) and one functional scale (dysphagia). Answers are expressed on scales ranging from 0 to 100 according to the EORTC scoring manual [9]. Higher scores in functional and global health status scales indicate a high or healthy level of functioning, whereas higher symptom scales indicate a high level of symptoms. Missing data were handled according to the EORTC scoring manual.

### Statistical analysis

Data were entered in Excel (Microsoft Office 365, Microsoft Corporation, Redmond, USA). Statistical analysis was performed using SPSS Statistics 23 (IBM Corporation, Armonk, USA). Due to the number of patients, the Mann–Whitney *U* test or Kruskal–Wallis test for all metric and Fisher’s exact test for all categorical variables were used. A *p*-value < 0.05 was considered statistically significant.

## Results

### Patients

Fifty-eight patients were included in the study. The study cohort consisted of 29 men and 29 women, with a mean age of 65 years at the time of diagnosis (range: 21–92). Most common cause of esophageal perforation was iatrogenic (*n* = 30, 51.7%), followed by spontaneous (*n* = 16, 27.6%) and traumatic perforation (*n* = 5, 8.6%). Further demographics and clinical details of patients are shown in Table 1. Based on primary treatment choice, patients were divided into three groups: endoscopic, surgical, and conservative.

**Table 1:** Demographic and clinical characteristics of 58 patients with esophageal perforation (non-follow-up and follow-up patients)

Variables	All (*n* = 58)	Non-follow-up (*n* = 38)	Follow-up (*n* = 20)	*p* value
	*n* (%)	*n* (%)	*n* (%)	
Gender, female	29 (50)	18 (47.4)	11 (55)	0.783
Age (years)	64.6 (SD 13.9)	64.9 (SD 15.1)	64 (SD 11.6)	0.600
BMI (kg/m^2^)	24.4 (SD 4.4)	24.6 (SD 4.1)	24.1 (SD 5.1)	0.404
Smoking	9 (15.5)	8 (21.1)	1 (5)	0.143
Alcohol	11 (19.0)	11 (28.9)	0 (0)	0.011
Comorbidity	55 (94.8)	36 (94.7)	19 (95)	1.000
Arterial hypertension	27 (46.6)	18 (47.4)	9 (45)	1.000
Atrial fibrillation	17 (29.3)	11 (28.9)	6 (30)	1.000
COPD	10 (17.2)	7 (18.4)	3 (15)	1.000
Coronary heart disease	10 (17.2)	9 (23.7)	1 (5)	0.141
Diabetes	7 (12.1)	6 (15.8)	1 (5)	0.403
Gastric and duodenal ulcers	6 (10.3)	3 (7.9)	3 (15)	0.405
Achalasia	4 (6.9)	1 (2.6)	3 (15)	0.114
Cause of perforation				
Iatrogen	30 (51.7)	19 (50)	11 (55)	0.787
Spontaneous	16 (27.6)	12 (31.6)	4 (20)	0.538
Traumatic	5 (8.6)	2 (5.3)	3 (15)	0.328
Other	4 (6.9)	3 (7.9)	1 (5)	1.000
Unknown	3 (5.2)	2 (5.3)	1 (5)	1.000
Site of perforation				
Proximal	10 (17.2)	2 (5.3)	8 (40)	0.002
Middle	21 (36.2)	14 (36.8)	7 (35)	1.000
Distal	27 (46.6)	22 (57.9)	5 (25)	0.026
Size of perforation (cm)	2 (0.4–11)	2 (0.4–11)	2 (1–6)	0.627
Delayed diagnosis > 24 h	20 (34.5)	11 (28.9)	9 (45)	0.362
Delayed diagnosis (days)	4.5 (2–35)	9 (2–35)	3 (2–7)	0.142

Categorical variables are expressed as number (percentage); continuous variables are expressed as mean (standard deviation)

**Fig. 1:**
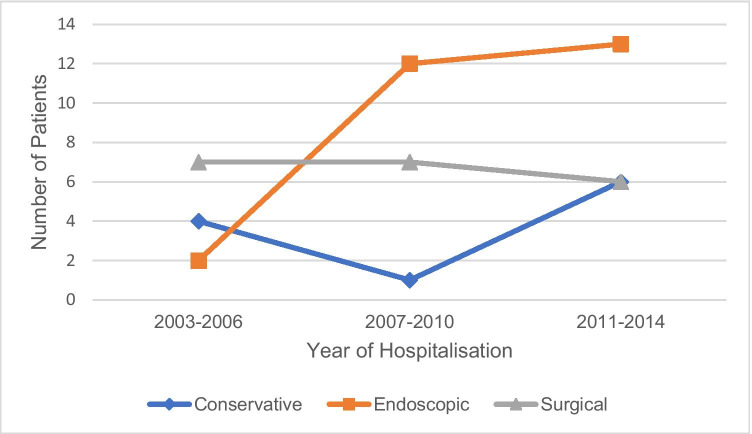
Development of treatment selection for esophageal perforation over time

With the upcoming endoscopic treatment options in the latest years, primary treatment selection shifted from surgical to mainly endoscopic therapy as shown in Fig. 1.

Twenty-seven patients (46.6%) were treated endoscopically. Esophageal stenting was performed in 18 patients with a median duration of stent placement of 33 days (range: 14–82). Stents were changed once on average, twice at the most. Two patients were treated with endoscopic vacuum therapy (EVT) with a median sponge inlay of 10.5 days and at least two endoscopic changes. Seven patients received a combined treatment of stent and EVT with a median stent inlay of 9 days (range: 4–50) and a median EVT of 10 days (range: 4–27) and a median sponge changing of 2 (range: 1–7).

Surgery was performed in 20 patients (34.5%). Fifteen patients initially received a transthoracic esophagectomy. Cervical diversion was required in 13 of these patients (65%). In 7 patients, gastrointestinal reconstruction was performed later on. The remaining 5 patients were treated with primary suture of the perforated area. In three of these patients, transthoracic esophagectomy was performed in the follow-up (2 × cervical deviation, 1 × intrathoracic esophagogastrostomy). Median time to reconstruction of cervical esophagostomy was 11.5 months (range: 6–42).

Conservative management was applied in 11 patients (19%). Therapy mainly consisted of antibiotics, proton pump inhibitors, parenteral nutrition, and in 5 cases chest tube drainage.

### Outcome

Outcome parameters were compared between treatment groups to identify possible differences. Overall, median hospital stay was 31.5 days (range 6–243), and median ICU stay was 9 days (range 1–200). Both were significantly longer in patients treated with surgery (*p* = 0.017; 0.003). Patients suffered from various complications including pleural effusion and empyema or pneumonia. Complication rate was highest in the surgical group with 85%, compared to 72.7% (*n* = 8) in the conservative and 81.5% (*n* = 22) in the endoscopic group. Even though no statistical difference could be demonstrated, thirteen patients had to return to the hospital for further therapy of late complications like esophageal stenosis, most of whom had been treated endoscopically (*n* = 8, 29.6%). Overall, 90-day mortality rate was 13.8% (*n* = 8, 4 endoscopic, 2 conservative, 2 surgical). Further details are displayed in Table 2.

**Table 2:** Short-term outcome stratified by treatment groups

Variables	All (*n* = 58)	Conservative (*n* = 11)	Endoscopic (*n* = 27)	Surgical (*n* = 20)	*p* value
	*n* (%)	*n* (%)	*n* (%)	*n* (%)	
Complications	42 (72.4)	7 (63.6)	18 (66.7)	17 (85)	0.279
Pleural effusion	33 (56.9)	7 (63.6)	16 (59.3)	10 (50)	0.781
Pneumonia	14 (24.1)	1 (9.1)	3 (11.1)	10 (50)	0.005
Pneumothorax	12 (20.7)	2 (18.2)	6 (22.2)	4 (20)	1.000
Pleural empyema	9 (15.5)	0 (0)	3 (11.1)	6 (30)	0.060
Sepsis	9 (15.5)	2 (18.2)	2 (7.4)	5 (25)	0.254
Hospital stay (days)	31.5 (6–243)	18 (8–54)	36 (6–89)	42.5 (18–243)	0.017
ICU stay (days)	9 (1–200)	4.5 (1–12)	9 (1–59)	21 (4–200)	0.003
Hospital readmission	13 (22.4)	1 (9.1)	8 (29.6)	4 (20)	0.424
In-hospital mortality	7 (12.1)	1 (9.1)	4 (14.8)	2 (10)	1.000
90-day mortality	8 (13.8)	2 (18.2)	4 (14.8)	2 (10)	0.789

Categorical variables are expressed as number (percentage), continuous variables are expressed as median (range)

### Health-related quality of life

At the onset of the study, 25 of 58 (43.1%) patients were already deceased. Of the remaining 33 patients, 20 were available for the survey. HRQoL was measured after a median follow-up of 49 months. Eight patients had been treated endoscopically, 6 had underwent surgery, and another 6 had been treated conservatively. Due to the small number of cases, we were not able to find statistically significant results, except for a single symptom scale in the EORTC QLQ-C30.

The study found a general trend of lower HRQoL in patients with non-neoplastic esophageal perforation compared to published reference data [10]. The endoscopic treatment group showed the highest GIQLI overall score and highest EORTC general health status, followed by the conservative and the surgical group, although the order varied between subscales. Surgical patients generally showed the lowest HRQoL scores.

**Fig. 2:**
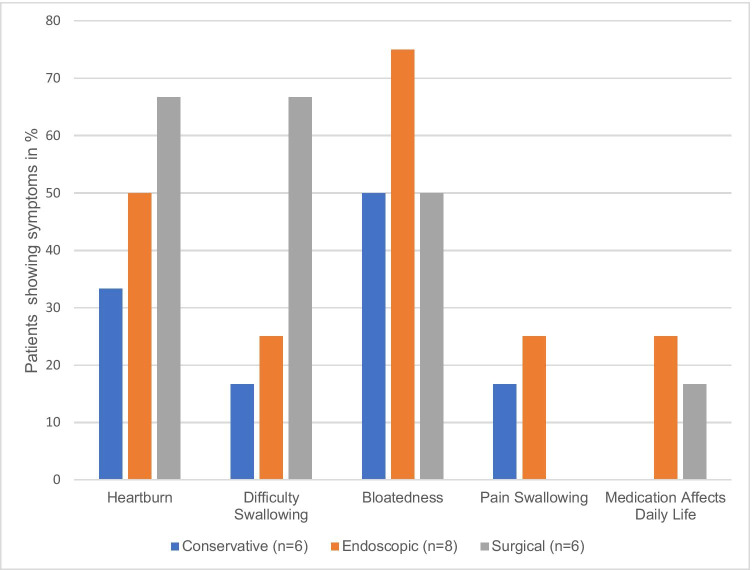
Relative frequencies of symptoms in follow-up patients assessed by the HRQL for GERD. Scores > 0 were classified as symptoms

Mean overall score for HRQoL index for GERD was 4.60 (SD 4.3). The surgical treatment group had the highest overall score with 6.2 (SD 4), indicating severe symptoms, followed by the endoscopic (4.4, SD 4.5) and the conservative group (3.3, SD 4.6). Sixty percent (*n* = 12) of follow-up patients were generally satisfied with their current health status. Only three patients showed no symptoms. The other patients reported mainly a feeling of fullness (60%, *n* = 12) and heartburn (50%, n = 10) (Fig. 2).

**Fig. 3:**
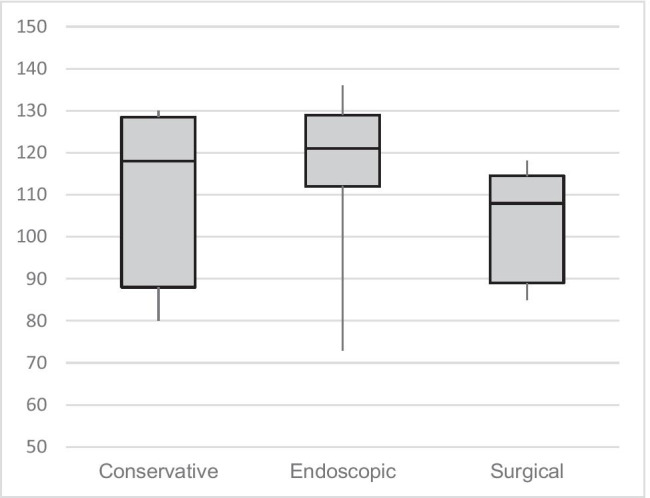
Boxplots for overall GIQLI scores stratified by treatment groups (conservative *n* = 6, endoscopic *n* = 8, surgical *n* = 6). Higher scores indicate better quality of life

Results of GIQLI showed a mean overall score of 111 (SD 18.9) compared to a reference score for healthy individuals of 125.8 (7). Most problems occurred in the physical function domain: 75% (*n* = 15) of patients reported tiredness and 65% (*n* = 13) woke up 3–4 nights of the week. All subdomains showed highest scores for the endoscopic treatment group and lowest scores for the surgical group (Fig. 3).

**Fig. 4:**
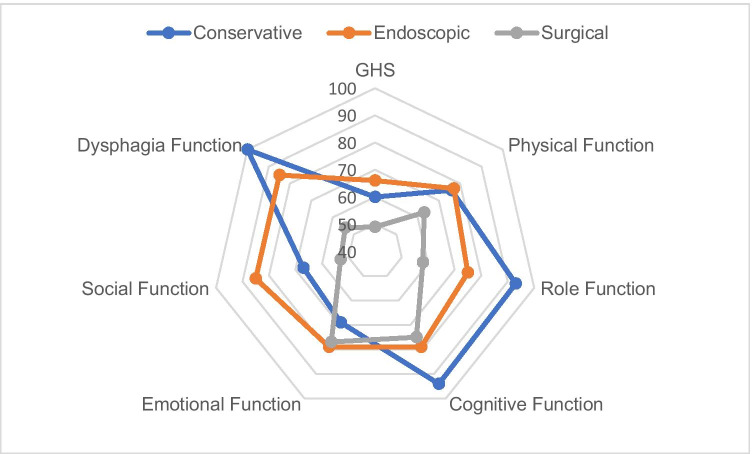
Global Health Score (GHS) and functional scales of the EORTC QLQ-C30 and QLQ-OES18 stratified by treatment groups (conservative *n* = 6, endoscopic *n* = 8, surgical *n* = 6). Higher scores indicate higher quality of life or higher level of function

Global health status (GHS) in the EORTC QLQ-C30 was lower for patients with non-neoplastic esophageal perforation than healthy individuals. Follow-up patients reached a mean score of 58.8 (SD 25.4) compared to 71.2 (SD 22.4) in the reference population [10]. Only two scores showed a statistical difference between treatment groups: Insomnia was reported by all conservatively treated patients (100%, n = 6) vs. 25% of endoscopic patients (n = 2) and constipation was reported exclusively by conservatively treated patients. Common symptoms were fatigue (84.2%, n = 16) and insomnia (60%, n = 12), matching the results of the GIQLI. The QLQ-C30 and QLQ-OES18 found lower function scores and higher symptom scores for the surgical group compared to conservatively or endoscopically treated patients. The GHS and functional scores are shown in Fig. 4.

## Discussion

In the immediate period after esophageal perforation, especially in malignant disease, morbidity and mortality are most essential. Yet, in benign, non-neoplastic disease with normal life expectancy after successful treatment, long-term quality of life becomes the most substantial focus. Therefore, the present study was conducted to report long-term HRQOL after successful therapy of non-neoplastic esophageal perforation. In addition, the study evaluated differences between treatment modalities regarding HRQOL in the short- and long-term follow-up.

The morbidity and mortality rate in our study is consistent with previously published studies of non-neoplastic esophageal perforation. In recently published meta-analyses and studies, mortality ranges from 12 to 28% [1, 11, 12]. Our mortality rate of 13.8% is at the lower end of published data. The median length of hospital stay was 31.5 in our patient group and 32.9 days reported by Biancari et al. [12].

The complication rate in our series is substantially higher than previously published data [13, 14]. Yet, we were able to demonstrate a lower rate of severe complications such as sepsis (15.5%). The most common complications in our series were pulmonary complications such as pleural effusion and pneumonia. This is consistent with large prospective studies on complications after esophagectomy [15, 16].

Notably and in contrast to previously published data, endoscopic treatment did not turn out to be superior to other treatment modalities in regard to short-term morbidity and mortality. Endoscopic treatment in fact led to the highest mortality in our series which in our hypothesis could possibly be caused by our low overall mortality of 13.8% [12–14, 17–19].

### Health-related quality of life

We were able to show that HRQoL was generally lower in patients with non-neoplastic esophageal perforation compared to a healthy reference population. However, the reported symptoms causing the lower HRQoL were not primarily related to esophageal dysfunction, raising the question whether the used questionnaires were able to reflect HRQoL associated with esophageal function properly. Comparing HRQoL results between the different treatment groups, the study shows a trend in favor of non-operative treatment. Endoscopic treatment seems to result in better HRQoL than conservative or surgical therapy. These findings were consistent for the GIQLI and the EORTC questionnaires. Due to the small patient cohort, these results are not statistically significant. Comparing long-term follow-up, a clear confounding factor is treatment selection based on underlying complications and comorbidities. Old, multi-morbid patients for example might more often be treated conservatively, while severe mediastinitis might lead to immediate surgery.

Several studies investigate HRQoL after esophageal surgery for malignancy. We have previously reported HRQoL after esophagectomy and transhiatal gastrectomy in patients with carcinoma of the gastroesophageal junction and were able to show that patients after esophagectomy exhibited a decreased HRQoL compared to patients after gastrectomy [20]. Yet compared to our data, EORTC questionnaires of patients after esophagectomy for cancer demonstrated a better HRQoL than surgically treated patients for perforation. Likewise, the mean Global Health Score reported by Fuchs et al. was 60.0 compared to 48.6 in our data. This superiority was consistent across subscales for physical function, role function, and emotional and social function. Furthermore, fatigue, nausea, pain, dyspnea, restlessness, and financial problems were more common in our data. This result is surprising considering the malignant nature of the underlying disease in the study by Fuchs et al.

Young et al. reported HRQoL in 81 patients with esophagectomy for benign disease such as motility disorders, esophageal strictures, hiatal hernia, and 6 patients with perforation of the esophagus using the SF-36 questionnaire [21]. With a median follow-up of 9.8 years, patients after esophagectomy showed an inferior general health status as well as physical and social function compared to general population. Keeping in mind the divergent questionnaires, this finding matches our data for surgically treated patients.

Dhayat et al. compared HRQoL of patients after endoscopic vacuum treatment for esophageal perforation or anastomotic leakage after esophagectomy and gastrectomy with patients without leakage [22]. HRQoL was evaluated with the GIQLI questionnaire with a median follow-up of 20 months. They reported a mean GIQLI score of 83 points compared to 116 points in the endoscopically treated group in our study. The superior result in our group is likely due to a higher proportion of patients with malignant disease and postoperative complications in the study by Dhayat et al.

A thorough literature review revealed three publications that exclusively focused on HRQoL after non-neoplastic esophageal perforations. Varghese et al. published a case report of four cases with reduced HRQoL after esophageal resection due to Boerhaave syndrome [23]. They only used a basic 1–10 score to evaluate HRQoL without validated questionnaires. A Norwegian group published two papers on HRQoL after esophageal perforation. One study covers bolus impactation (*n* = 10), while the other deals with iatrogenic perforations (*n* = 5) [24, 25].

All three publications are limited by small sample size. Furthermore, patient selection (surgically treated patients only, iatrogenic perforation, bolus impactation) is a confounding factor. Our study is the first to include endoscopically placed vacuum therapy in the endoscopic treatment regimen and compares outcome between different treatment modalities. In summary, in this study, we present the largest patient cohort with a thorough evaluation with the use of validated questionnaires and a direct comparison of HRQoL in different treatment modalities.

### Strengths and limitations

Even though the present study is the largest observational study regarding diagnosis and number of patients, a limitation is the low number of cases within different treatment groups. This makes extensive statistical analyses difficult as well as drawing evidence-based recommendation for general clinical management.

Additional limitation is specific to the utilized questionnaires. So far, no standardized, validated, and especially designed questionnaire for patients treated for non-neoplastic esophageal perforation is available. Therefore, we used the EORTC and the GIQLI score which are commonly used to assess quality of life in upper GI patients [26]. We found that the results for the EORTC and the GIQLI score were comparable. Yet, the symptoms that lead to the diminished HRQoL were mainly not related to the esophagus. Therefore, signs of esophageal dysfunction could be underestimated in our cohort. We do believe that the use of symptom scores such as the EORTC and QLQ-C30 are advantageous, as it allows the comparison of HRQOL data to a large reference population.

Another point that must be discussed and limits the relevance of this study is its retrospective character. Due to the emergency treatment situation, we were not able to compare our data to pretreatment baseline HRQOL. A significant part of primarily successfully treated patients was already deceased at the time of questioning, and information on patients’ cause of death were not available. Yet, considering the fact that the discussed disease always calls for an emergency treatment, prospective studies are unavailable and can hardly be expected in the future.

A strength of this study was the very strict inclusion criteria resulting in a homogenous patient cohort with benign, non-neoplastic disease only. Thus, results are not confounded by quality of life changes through carcinoma. With objective and validated questionnaires, data is compared to the normal population. Treatment was performed at a high-volume center for esophageal surgery with a vast experience of treating esophageal perforation and anastomotic leakage. This allowed immediate and optimal treatment based on individual patient needs and not on availability of caregivers.

## Conclusion

In this study, our historical collective was analyzed and described. In our experience, esophageal perforation ought to be treated fast and aggressively in order to minimize morbidity and mortality. Quality of life in patients with non-neoplastic esophageal perforation is still reduced in the long-term follow-up, but reported symptoms are not imperatively caused by the event of esophageal injury. New emerging endoscopic treatments proved to be a good alternative to the well-established surgical options [19]. There seems to be a trend towards endoscopic treatment. Since more endoscopic treatment was performed in the later phase of the collective, no clear recommendation for treatment can be made. We would like to emphasize that endoscopic treatment should always be seen as an option. Since the endoscopic treatment rarely seems to fail saving patients from esophagectomy, it should always be considered a valid primary treatment option, especially when one considers that short-term complication rates and the length of ICU stays as well as hospital stays in general were considerably higher after operative treatment.

## Data Availability

The datasets during and/or analyzed during the current study are available from the corresponding author on reasonable request.
